# Serum soluble PD‐1 plays a role in predicting infection complications in patients with acute pancreatitis

**DOI:** 10.1002/iid3.394

**Published:** 2021-01-08

**Authors:** Xingxing Yu, Yu Pan, Qinglin Fei, Xianchao Lin, Zhijiang Chen, Heguang Huang

**Affiliations:** ^1^ Department of General Surgery Fujian Medical University Union Hospital Fuzhou China

**Keywords:** acute pancreatitis, immunosuppression, infection complication, PD‐1, PD‐L1

## Abstract

**Background:**

Most of acute pancreatitis (AP) are mild and self‐limiting, however, 15%–20% of patients develop severe acute pancreatitis (SAP) or moderately acute pancreatitis (MSAP) with local or systemic complications. Infection complications (ICs) result in 40%–70% morbidity and high mortality rates among SAP and MSAP patients. It is more important to early recognize of ICs of MSAP or SAP. Several studies have indicated that serum soluble programmed cell death protein (sPD‐1) or programmed cell death 1 ligand (sPD‐L1) levels were higher in patients with severe sepsis than in healthy volunteers and have a predictive capacity for mortality. However, the role of serum sPD‐1/sPD‐L1 in AP remains unclear. This study aimed to investigate whether the ICs of AP patients is associated with their sPD‐1 and sPD‐L1 levels, which were determined via enzyme‐linked immunosorbent assay of peripheral blood samples from 63 MSAP and SAP patients and 30 healthy volunteers.

**Results:**

The serum sPD‐1 levels in AP patients on Days 1, 3, and 10 after onset were significantly increased in a time‐dependent manner compared with that in healthy volunteers. Moreover, the AP patients with ICs had significantly higher serum sPD‐1 levels than the AP without ICs. While serum sPD‐L1 levels in AP were similar to that in healthy volunteers. Besides, serum levels of sPD‐1/sPD‐L1 were negatively correlated with circulating lymphocytes. Univariate and multivariate regression analyses showed that the upregulated serum sPD‐1 level was an independent risk factor for ICs in AP. The area under the receiver operating characteristics curve indicated that combination with Acute Physiology and Chronic Health Evaluation II score and serum sPD‐1 level had a high accuracy in predicting ICs in AP.

**Conclusion:**

Serum sPD‐1/sPD‐L1 may be involved in the immunosuppressive process in AP. Moreover, the serum sPD‐1 level may be an independent risk factor for predicting ICs in AP patients.

## Graphical abstract

This study investigated whether the immune status and infection complications of acute pancreatitis (AP) patients are associated with their serum soluble programmed cell death protein (sPD‐1) or programmed cell death 1 ligand (sPD‐L1) levels. We believe that our study makes a significant contribution to the literature because it clearly demonstrates that serum sPD‐1/sPD‐L1 may be involved in the immunosuppressive process in AP, and the serum sPD‐1 level may be an independent risk factor for predicting ICs in AP patients.

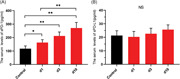

**Abbreviations**
AP: acute pancreatitis
APACHE II: Acute Physiology and Chronic Health Evaluation II
BISAP: Bedside Index for Severity in Acute Pancreatitis
HCT: hematocrit
IQR: interquartile range
LMR: lymphocyte–monocyte ratio
MSAP: moderately severe acute pancreatitis
NLR: neutrophil–lymphocyte ratio
PLR: platelet–lymphocyte ratio
SAP: severe acute pancreatitis
sPD‐1: serum soluble programmed cell death
sPD‐L1: serum soluble programmed cell death protein ligand 1
WBC: white blood cell

## 1 BACKGROUND

Acute pancreatitis (AP) is a common acute abdomen in general surgery, and most of the APs are mild and self‐limiting, without complications and only needing a short hospitalization.^1^ However, 15%–20% of patients develop severe acute pancreatitis (SAP) or moderately severe acute pancreatitis (MSAP) with local or systemic complications, which has a high mortality.^2^, ^3^ The main reason for the high mortality among SAP and MSAP patients is the infection complications (ICs), morbidity for which can be approximately 40%–70%.^4^, ^5^, ^6^ It's required that early identification of SAP and MSAP patients at risk of developing ICs.

Studies showed that early immunosuppression of SAP has led to the occurrence of systemic ICs and multiple organ failure.^7^, ^8^ Programmed cell death protein (PD‐1) is a co‐inhibitory molecule belonging to the CD28 family, mainly expressed in activated T lymphocytes, natural killer T cells, and bone marrow cells.^9^, ^10^ The programmed cell death 1 ligand (PD‐L1) is a ligand for PD‐1 expressed on antigen‐presenting and hematopoietic cells.^9^ The PD‐1/PD‐L1 pathway has been shown to regulate lymphocyte proliferation and apoptosis and play an important role in immune regulation.^11^, ^12^, ^13^ Previous studies had shown that PD‐1 and PD‐L1 exist in two forms: cell membrane‐bound and soluble forms.^14^ Soluble PD‐1 and PD‐L1 (sPD‐1/sPD‐L1) can be detected in human serum. sPD‐1 may promote T‐cell responses by inhibiting the PD‐1/PD‐L1 signaling pathways, while excessive sPD‐1 may lead to immunosuppression^14^; sPD‐L1 was released into the blood by the surface of PD‐L1‐expressing cells that may reflect PD‐L1 levels.^15^ Additionally, sPD‐L1 may retain immunosuppression induction.^15^ A recent study revealed that PD‐1 expression in peripheral T cells and PD‐L1 expression in monocytes increased significantly in sepsis patients than in healthy controls,^16^ and in AP patients with ICs than the patients without ICs.^17^ Several studies have indicated that serum sPD‐1/sPD‐L1 levels were higher in patients with severe sepsis than in healthy volunteers and had a predictive capacity for mortality.^18^, ^19^ However, the relationship between serum sPD‐1/sPD‐L1 levels and ICs in AP has not been certified. Furthermore, serum sPD‐1/sPD‐L1 expression is easy to examine and has potential applications.

In this study, we investigated the levels of serum sPD‐1/sPD‐L1 in SAP and MSAP patients and healthy volunteers to understand the association of these parameters with immune status and ICs in AP patients.

## 2 METHODS

Peripheral blood was obtained from 63 patients with MSAP or SAP at Fujian Medical University Union Hospital, Fuzhou, China, from October 2017 to April 2019. Patient inclusion criteria included: (1) patients with MSAP or SAP, according to the 2012 edition of the Atlanta Convention AP classification criteria^20^; (2) aged 18 years or older; (3) admitted to the hospital within 48 h of onset. Exclusion criteria included: patients (1) with mild AP; (2) treated for <10 days; (3) with chronic pancreatitis, pregnancy, breastfeeding, acute and chronic hepatitis, end‐stage liver and kidney disease, immunodeficiency disease, and malignant tumor; (4) who had received immunosuppressive therapy. All patients were followed until discharge or hospital mortality. Patient baseline characteristics, Bedside Index for Severity in Acute Pancreatitis (BISAP), Ranson, and Acute Physiology and Chronic Health Evaluation II (APACHE II) scores were collected and recorded. Patient characteristics were collected and are shown in Table 1. This study was approved by the Committee for the Ethical Review of Research, Fujian Medical University Union Hospital.

**Table 1:** Characteristics of AP patients

Characteristics	Data (*n* = 63)
Age (years, mean ± *SD*)	51.08 ± 13.56
Sex (*n*, female/male)	29/34
Severity of AP, *n* (%)	
Moderately severe	35 (55.6)
Severe	28 (44.4)
Etiology of AP, *n* (%)	
Biliary	19 (30.2)
Hypertriglyceridemia	24 (38.1)
Alcoholicity	4 (6.3)
Other	16 (25.4)
Ranson score, median (IQR)	2.0 (1–3)
BISAP score, median (IQR)	2.0 (1–2)
APACHE II score, median (IQR)	10.0 (8–15)
Infection complications, *n*	
Pneumonia	36
Infected necrosis	20
Bacteremia	3
Organ dysfunction, *n*	
Respiratory	29
Cardiovascular	10
Renal	11
Interventions, *n*	
Surgical	29
Mechanical ventilation	7
Renal replacement therapy	3
Hospital mortality, *n* (%)	2 (3.2)

Abbreviations: AP, acute pancreatitis; APACHE II, Acute Physiology and Chronic Health Evaluation II; BISAP, The Bedside Index for Severity in Acute Pancreatitis; IQR, interquartile range.

Definition of ICs: infected pancreatic necrosis, bacteremia, pneumonia, infectious ascites, or urinary tract infections during admission. The diagnostic criteria for infected pancreatic necrosis were “positive for peripancreatic effusion or pancreatic necrosis tissue culture” obtained at the first pancreatic perivascular drainage or the first surgical treatment. The diagnostic criterion for bacteremia was “positive for blood culture.” Diagnostic criteria for pneumonia included: (1) newly developed cough, or symptoms of the original respiratory disease, with purulent sputum, with or without chest pain; (2) fever ≥38°C; (3) lung consolidation signs and/or wet rales; (4) white blood cell (WBC) >10 × 10^9^/L or <4 × 10^9^/L with or without nuclear left shift; (5) lung imaging suggests patchy infiltrating shadow or interstitial changes with or without pleural effusion. Any of the above (1) to (4) plus the fifth item can lead to a diagnosis, except for tuberculosis, lung cancer, noninfectious pulmonary interstitial disease, pulmonary edema, atelectasis, pulmonary embolism, pulmonary eosinophilic infiltration, and pulmonary vasculitis. The diagnostic criterion for infectious ascites is the positive ascites specimen obtained during the first abdominal puncture drainage or the first surgery. Diagnostic criteria (and confirmation) for urinary tract infections included: bacterial colony count ≥10^5^/ml and WBC count >10/HP following centrifugation of urine collected midstream. Multiple infections in the same patient were considered one endpoint.^17^

### 2.1 Blood samples

Peripheral blood samples were obtained from 30 healthy volunteers (control) and AP patients on Days 1, 3, and 10 after admission. Serum samples were collected immediately after centrifugation at 3000 rpm for 15 min at 4°C, and stored at −80°C for subsequent analysis.

### 2.2 Serum sPD‐1 and sPD‐L1 analysis

Serum sPD‐1/sPD‐L1 was quantified using the human sPD‐1/sPD‐L1 enzyme‐linked immunosorbent assay (ELISA) kit (RayBio®). Serum sPD‐1/sPD‐L1 levels were measured in duplicates and analyzed according to manufacturers' recommendations. A 1:50 dilution was used for all the samples. The nonlinear standard curve was constructed based on polynomial regression (degree = 2).

### 2.3 Statistical analysis

SPSS 22.0 software (SPSS Inc.) was used for statistical analysis. Results are presented as medians and interquartile ranges (IQRs) or mean ±*SD*, and categorical variables are shown as frequency and percentage. The normal distribution of all variables was tested using the Shapiro–Wilk test. *χ* ^2^ or Fisher's tests was used for two‐category variables. The independent sample *t* test was used to compare variables that conform to the normal distribution, and the Mann–Whitney *U* test to compare variables that are not normally distributed. The correlation was assessed by a Spearman rank test. The concentrations at different times (Days 1, 3, and 10) in each group were compared using one‐way repeated measures analysis of variance. A two‐category univariate logistic regression analysis was performed to assess the correlation between the variables (Table 2) and AP infectious complications. Then only the significant differences in univariate analysis were using multivariate stepwise regression analysis of variables. The area under the receiver operating characteristics (ROCs) curve (AUC) was used to estimate the accuracy of the predicted model, and the AUC was bilaterally *p* < .05. Figures were prepared using GraphPad Prism version 6.0 (GraphPad Software).

**Table 2:** Univariate and multivariate regression analysis of variables for ICs of AP

	Univariate analysis		Multivariate analysis	
Variable	OR (95% CI)	*p* value	OR (95% CI)	*p* value
Age	1.016 (0.979–1.055)	.395		
HCT (%)	0.908 (0.842–0.980)	**.013**	0.921 (0.842–1.007)	.071
APACHE II score	1.420 (1.134–1.776)	**.002**	1.281 (1.008–1.629)	**.043**
Lymphocyte count on Day 1	0.863 (0.380–1.957)	.724		
Monocyte count on Day 1	3.178 (0.757–13.344)	.114		
Neutrophil count on Day 1	0.987 (0.898–1.084)	.783		
Serum sPD‐1 levels on Day 1	1.002 (0.997–1.007)	.428		
Serum sPD‐1 levels on Day 3	1.013 (1.005–1.021)	**.002**	1.009 (1.001–1.018)	**.029**
Serum sPD‐L1 levels on Day 1	0.985 (0.959–1.013)	.300		
Serum sPD‐L1 levels on Day 3	1.005 (0.976–1.063)	.722		

*Note*: Bold values represent factors greater than .05 in the *p* value.

Abbreviations: AP, acute pancreatitis; APACHE II, Acute Physiology and Chronic Health Evaluation II; CI, confidence interval; HCT, hematocrit; IC, infection complication; OR, odds ratio.

## 3 RESULTS

### 3.1 Characteristics of the patients

According to the revised Atlanta classification,^20^ a total of 63 patients with AP (28 SAP and 35 MSAP patients) were included in this study, with an average age of 51.08 ± 13.56 years. For the classification of AP etiology, hypertriglyceridemia‐induced pancreatitis was the main cause, accounting for 38.1%, followed by biliary 30.2%, alcoholicity 6.3%, and other factors 25.4%. All AP patients underwent three AP‐related scoring system after admission, including the BISAP (assessment after 24‐h admission), Ranson (48‐h), and APACHE II (48‐h) scores. The clinical characteristics of these patients are shown in Table 1.

### 3.2 Serum sPD‐1 and sPD‐L1 levels in patients of AP

**Figure 1:**
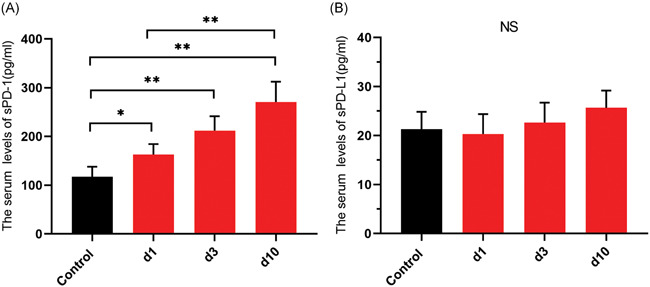
The serum sPD‐1 and sPD‐L1 levels in patients with acute pancreatitis (AP). (A) sPD‐1 and (B) sPD‐L1 were measured in peripheral blood from healthy volunteers (control, *n *= 30) and patients with AP (*n* = 63) on Day 1 (d1), Day 3 (d3), and Day 10 (d10) after onset. *p < .05, **p < .01

Serum levels of sPD‐1 and sPD‐L1 were measured in patients of AP on Day 1 (d1), Day 3 (d3), and Day 10 (d10) after admission. The serum sPD‐1 levels in AP patients on d1, d3, and d10 were significantly elevated compared with that in healthy controls (*p* < .05, *p* < .01, *p* < .01; Figure 1A). Moreover, serum sPD‐1 level in AP patients was upregulated in a time‐dependent manner, and was most elevated on Day 10 compared with that on Day 1 (*p* < .01; Figure 1A). However, serum sPD‐L1 levels on d1, d3, and d10 in AP patients were similar to that in healthy controls (Figure 1B).

### 3.3 Correlation between clinical indicators and serum sPD‐1/sPD‐L1 levels

We further investigated the relationship between clinical indicators and serum sPD‐1/sPD‐L1 levels. We observed that the serum levels of sPD‐1 and sPD‐L1 on Day 10 were both negatively correlated with lymphocyte count (*r* = −.335, *p* = .015; *r* = −.294, *p* = .035; Table 3), whereas the serum level of sPD‐1 on Day 1 was positively correlated with lymphocyte–monocyte ratio (LMR; *r* = .269, *p* = .034; Table 3). Moreover, the serum level of sPD‐1 on Days 3 and 10 was negatively associated with the hematocrit (HCT; *r* = −.289, *p* = .021; *r* = −.331, *p* = .016).

**Table 3:** Correlation between traditional clinical indicators and serum sPD‐1/sPD‐L1

		Day 1		Day 2		Day 3	
Variable		sPD‐1	sPD‐L1	sPD‐1	sPD‐L1	sPD‐1	sPD‐L1
Lymphocyte count (×10^9^/L)	*r*	.168	.156	−.092	−.023	**−.335**	**−.294**
	*p*	.188	.244	.474	.861	**.015**	**.035**
WBC (×10^9^/L)	*r*	.048	−.029	−.088	−.032	.133	.060
	*p*	.710	.820	.493	.800	.343	.638
Monocyte count (×10^9^/L)	*r*	−.068	−.144	−.049	−.040	−.211	−.080
	*p*	.595	.259	.703	.756	.134	.574
Neutrophil count (×10^9^/L)	*r*	−.162	−.052	−.051	−.085	−.038	.143
	*p*	.205	.686	.694	.507	.790	.312
HCT (%)	*r*	−.145	.133	**−.289**	.125	**−.331**	.116
	*p*	.829	.298	**.021**	.329	**.016**	.365
NLR	*r*	−.248	−.138	.038	−.024	.177	.213
	*p*	.061	.280	.766	.850	.210	.129
LMR	*r*	.169	**.268**	−.015	.019	−.049	−.092
	*p*	.185	**.034**	.910	.880	.728	.518
PLR	*r*	−.202	−.116	.035	−.046	.118	.068
	*p*	.113	.367	.787	.718	.404	.630

*Note*: Bold values represent factors greater than .05 in the *p* value.

Abbreviations: HCT, hematocrit; LMR, lymphocyte–monocyte ratio; NLR, neutrophil–lymphocyte ratio; PLR, platelet–lymphocyte ratio; WBC, white blood cell.

### 3.4 Correlation between serum sPD‐1/sPD‐L1 levels and ICs of AP

To investigate the relationship between ICs of AP and serum sPD‐1/sPD‐L1 levels, all patients were divided into two groups: AP with (*n *= 36) and without (*n *= 27) ICs. We found that APACHE II scores were significantly higher in the AP with ICs group than in the AP without ICs group (*p* < .001; Table 4). Whereas the HCT was significantly higher in the AP without ICs group than in the AP with ICs group (*p* = .003; Table 4). The AP with ICs group had significantly higher serum sPD‐1 levels on Days 3 and 10 than the AP without ICs group (*p* < .001, *p* < .001; Table 4). However, there were no significant differences between AP with ICs group and AP without ICs group with regard to serum sPD‐L1 levels.

**Table 4:** Clinical indicators of patients with AP with or without ICs

	AP with IC (*n* = 36)	AP without IC (*n *= 27)	*p* value
Age (years)	52.33 ± 13.76	49.41 ± 13.37	.436
Male/female (*n*)	24/12	10/17	–
APACHE II score	13.56 ± 2.91	8.93 ± 2.43	**<.001**
WBC count (×10^9^/L)	12.42 ± 5.41	12.97 ± 5.67	.755
Neutrophil count on Day 1 (×10^9^/L)	9.98 ± 5.23	10.35 ± 5.54	.824
Monocyte count on Day 1 (×10^9^/L)	0.84 ± 0.47	0.62 ± 0.30	.071
Lymphocyte count on Day 1 (×10^9^/L)	1.20 ± 0.58	1.26 ± 0.66	.760
PLT (×10^9^/L)	282.06 ± 116.47	239.44 ± 105.32	.090
HCT (%)	31.66 ± 7.79	36.79 ± 6.75	**.003**
sPD‐1 levels on Day 1 (pg/ml)	186.29 ± 124.51	165.57 ± 62.25	.890
sPD‐1 levels on Day 3 (pg/ml)	266.03 ± 130.37	185.17 ± 78.79	**<.001**
sPD‐1 levels on Day 10 (pg/ml)	323.76 ± 167.25	210.97 ± 102.33	**<.001**
sPD‐L1 levels on Day 1 (pg/ml)	25.83 ± 16.01	30.78 ± 21.51	.413
sPD‐L1 levels on Day 3 (pg/ml)	31.02 ± 17.61	29.49 ± 16.63	.890
sPD‐L1 levels on Day 10 (pg/ml)	27.58 ± 15.02	33.75 ± 14.81	.060

*Note*: Bold values represent factors greater than .05 in the *p* value.

Abbreviations: AP, acute pancreatitis; APACHE II, Acute Physiology and Chronic Health Evaluation II; HCT, hematocrit; IC, infection complication; PLT, platelet; WBC, white blood cell.

### 3.5 Serum sPD‐1 may be an independent factor for predicting ICs in AP

To determine the predictive effect of age, APACHE II scores, HCT, and lymphocyte, monocyte, and neutrophil counts on Day 1, and serum sPD‐1 and sPD‐L1 levels on Days 1 and 3 for ICs, we performed a logistic regression analysis. Univariate analysis demonstrated that HCT (odds ratio [OR], 0.908; 95% CI, 0.842–0.980; *p* = .013), APACHE II score (OR, 1.420; 95% CI, 1.134–1.776; *p* = .002), and serum sPD‐1 level on Day 3 (OR, 1.013; 95% CI, 1.005–1.021; *p* = .002) were significantly associated with ICs of AP (Table 2). Furthermore, we performed multivariate analysis to evaluate HCT, APACHE II score, and serum sPD‐1 level on Day 3 as independent predictors of ICs. The results suggested that serum sPD‐1 levels on Day 3 (OR, 1.009; 95% CI, 1.001–1.018; *p* = .029) and the APACHE II score (OR, 1.281; 95% CI, 1.008–1.629; *p* = .043) were independent risk predictors of ICs in AP (Table 2).

**Figure 2:**
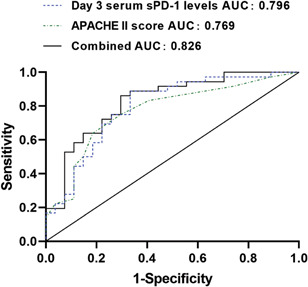
The area under the ROC curve (AUC) was used to estimate the accuracy of the predicted model. AUC of serum sPD‐1 level on Day 3: 0.796; AUC of Acute Physiology and Chronic Health Evaluation II score: 0.769; AUC of combined: 0.826

To evaluate the predictive accuracy of serum sPD‐1 levels on Day 3 and the APACHE II score for ICs in AP patients, the ROCs curve analysis was performed. The AUC values for serum sPD‐1 levels on Day 3 and APACHE II score were 0.796 (95% CI, 0.681–0.911; *p* < .001) and 0.769 (95% CI, 0.649–0.889; *p* < .001; Table 5 and Figure 2). By combining these two variables, a high accuracy for AP IC prediction was achieved (AUC = 0.826; 95% CI, 0.721–0.931; *p* < .001; Table 5 and Figure 2).

**Table 5:** AUCs of various parameters for predicting ICs in AP patients

Variable	AUC	*p* value	95% Cl
APACHE II score	0.769	<.001	0.649–0.889
Serum sPD‐1 levels on Day 3	0.796	<.001	0.681–0.911
Combination of above two various	0.826	<.001	0.721–0.931

Abbreviations: AP, acute pancreatitis; APACHE II, Acute Physiology and Chronic Health Evaluation II; AUC, area under the curve; CI, confidence interval; IC, infection complication.

## 4 DISCUSSION

MSAP and SAP could develop into immunosuppression, leading to secondary infection and pancreatic necrosis.^21^, ^22^ Our study showed that compared with healthy volunteers, serum sPD‐1 levels in the MSAP and SAP patients increased continuously during the early course of the disease, especially the patients with ICs. Moreover, elevated sPD‐1 level was associated with enhanced occurrence of ICs. Studies showed that serum sPD‐1 may promote T‐cell responses by inhibiting the PD‐1/PD‐L1 signaling pathway, but the continuously excessive level of serum sPD‐1 may serve as an antibody to block the PD‐1/PD‐L1 pathway, which leads to the aberrant activation and proliferation of T cells.^14^, ^23^ The uncontrolled immune regulation resulted in hyperimmune behavior in the early stage of SAP, however, with the consumption of lymphocytes, the hyperimmune status transformed into immunosuppression and increased the incidence of ICs. Finally, a marked increase in sPD‐1 levels may represent more severe immune damage in patients.^18^ In addition, sPD‐L1 may retain the immunosuppressive condition and continuously increased sPD‐L1 ultimately aggravates immunosuppression.^15^, ^24^ Hence, serum sPD‐1/sPD‐L1 levels may play an important role in monitoring the immune status of AP patients and predicting ICs and prognosis.

Furthermore, our data indicated that serum sPD‐1/sPD‐L1 levels of AP patients are associated with LMR, HCT, and lymphocyte counts. Immune dysfunction in AP patients may be caused by decreased peripheral blood lymphocytes.^24^ The decreased expression of human leukocyte antigen‐DR (HLA‐DR) on monocytes may lead to early immunosuppression of AP.^25^ Moreover, serum sPD‐L1 was reported to be involved in lymphocyte apoptosis.^15^ Our investigation of the correlation between serum sPD‐1/sPD‐L1 and clinical indicators revealed that dynamic monitoring of serum sPD‐1/sPD‐L1 levels in AP patients may reflect systemic immunologic functions in AP patients.

Previous studies indicated that BISAP, Ranson, and APACHE II scores could predict the mortality of AP patients with high accuracy^26^, ^27^, ^28^ and high Ranson, BISAP, and APACHE II scores were also associated with organ failure and complications in AP patients.^29^, ^30^ In this study, we showed that the elevated serum sPD‐1 level was an independent risk factor for ICs in patients of AP. Combination of APACHE II score and serum sPD‐1 level may better predict ICs of AP patients. Depending on the prediction of sPD‐1 in AP patients, the level of sPD‐1 in different grades may use to guide the prevention and treatment of ICs in AP patients. The previous study pointed out that the combination of anti‐inflammatory and immune‐stimulating therapies may be a new promising approach to AP immunotherapy.^31^ In the complex inflammatory response of AP, a large number of pro‐inflammatory cytokines such as TNF‐α and IL‐6 were produced and maintained the pro‐inflammatory–anti‐inflammatory balance process of AP. In addition, pro‐inflammatory cytokines, such as TNFα and IL‐6, could modulate the level of sPD‐1 in vitro and TNF‐inhibitor therapy could modulate sPD‐1 levels in the serum and synovial fluid of patients with inflammatory arthritis.^32^ Further study to reduce the production of sPD‐1 through anti‐pro‐inflammatory cytokines may enhance the immune status and reduce the occurrence of ICs. However, there remain some limitations in this study, we investigated these variates in AP patients from a single center and the number of cases in this study was small. Further study involving a large cohort from multiple centers is needed to confirm these results.

## 5 CONCLUSIONS

Serum sPD‐1/sPD‐L1 levels may be involved in the immunosuppressive process of AP, and sPD‐1, which increases continuously in the peripheral blood of AP patients, maybe an independent risk factor for predicting ICs in AP patients, which is potentially applicable in determining or improving AP patient prognosis.

## CONFLICT OF INTERESTS

The authors declare that there are no conflict of interests.

## ETHICS STATEMENT

All procedures performed in studies involving human participants were in accordance with the Helsinki declaration. All patients whose blood samples were used in this study provided written informed consent, and the study was approved by the Committee for the Ethical Review of Research, Fujian Medical University Union Hospital.

## AUTHOR CONTRIBUTIONS

Yu Pan, Xianchao Lin, and Heguang Huang conceived the concept. Heguang Huang supervised the study. Yu Pan, Qinglin Fei, Xingxing Yu designed and performed the experiments. Yu Pan, Xianchao Lin wrote the manuscript. All authors approved the manuscript.

## Data Availability

The data that support the findings of this study are available from the corresponding author upon reasonable request.
